# Supplementary material for: MetaRibo-Seq measures translation in microbiomes
Source: Nat Commun. 2020 Jun 29;11:3268. doi: 10.1038/s41467-020-17081-z (PMC7324362; doi:10.1038/s41467-020-17081-z)
Supplement: Supplementary file 10 — Supplementary Data 7 [file 41467_2020_17081_MOESM10_ESM.zip › File2/Confidence_High_Taxonomy/146171_out.krona.html]

Javascript must be enabled to view this page.

members
magnitude
magnitudeUnassigned
count
unassigned
taxon
rank

146171\_out

3

superkingdom
2
2

2
1239
phylum

186801
class
2

2
186802
order

family
541000
1

1
1263
genus

species
2292374
1

SRS146888\_contig\_number\_contig-100\_4601.4602

1
family
186803

1
1769710
genus

1
1653434
species

SRS097889\_contig\_number\_19410

1

SRS146888\_contig\_number\_contig-100\_10143.82395
